# Draft Genome Sequence of a Sulfide-Oxidizing, Autotrophic Filamentous Anoxygenic Phototrophic Bacterium, *Chloroflexus* sp. Strain MS-G (*Chloroflexi*)

**DOI:** 10.1128/genomeA.00872-14

**Published:** 2014-09-04

**Authors:** Vera Thiel, Trinity L. Hamilton, Lynn P. Tomsho, Richard Burhans, Scott E. Gay, Stephan C. Schuster, David M. Ward, Donald A. Bryant

**Affiliations:** a Department of Biochemistry and Molecular Biology, The Pennsylvania State University, University Park, Pennsylvania, USA; b Department of Geosciences, Penn State Astrobiology Research Center (PSARC), The Pennsylvania State University, University Park, Pennsylvania, USA; c Singapore Center on Environmental Life Sciences Engineering, Nanyang Technological University, Singapore; d Department of Chemistry and Biochemistry, Montana State University, Bozeman, Montana, USA; e Department of Land Resources and Environmental Sciences, Montana State University, Bozeman, Montana, USA

## Abstract

The draft genome sequence of the thermophilic filamentous anoxygenic phototrophic bacterium *Chloroflexus* sp. strain MS-G (*Chloroflexi*), isolated from Mushroom Spring (Yellowstone National Park, WY, USA) was sequenced and comprises 4,784,183 bp in 251 contigs. The draft genome is predicted to encode 4,059 protein coding genes, 49 tRNA encoding genes, and 3 rRNA operons.

## GENOME ANNOUNCEMENT

*Chloroflexus* sp. strain MS-G is a thermophilic, filamentous anoxygenic bacterium of the family *Chloroflexaceae* of the phylum *Chloroflexi*. Strain MS-G was isolated from a phototrophic microbial mat in an effluent channel of Mushroom Spring, an alkaline siliceous hot spring in the Lower Geyser Basin of Yellowstone National Park (44°32′20.4″ N, 110°47′52.8″ W, WY, USA). The 16S rRNA sequence of this isolate shares >99% nucleotide identity to a 16S rRNA sequence obtained from the metagenome (IMG taxon OID 3300002510) of the green upper layer (upper ~1 mm), and both share 100% with the most abundant *Chloroflexus* sp. ITAG sequence (253 bp) of the microbial mat at 60°C. The 16S rRNA sequence was 98% similar to cultured isolates *Chloroflexus* spp. strains 396-1 and NPE (EMBL acc. nos. AJ308498 and AJ308502 [1]), and ≤94% identical to that of the type strains *Chloroflexus aurantiacus* J-10-fl ^T^and *C. aggregans* DSM 9485^T^.

Purified genomic DNA of *Chloroflexus* sp. strain MS-G was sequenced using an Illumina MiSeq instrument. The draft genome was assembled with Newbler (version 2.9, Roche) from 2,715,946 paired end reads with a minimum length of 301 bp into 263 contigs. For genome analysis contigs without any predicted ORFs were excluded leading to an assembly of 251 contigs with a minimum length of 500 bp, comprising 4,784,183 bp with an average G+C-content of 55% and median coverage of 262-fold.

Annotation using RAST (2) predicted 4,064 protein coding genes and 49 tRNA-encoding genes, which is the same number of tRNA genes found in the *C. aurantiacus* strain J-10-fl^T^ genome (3). Based on coverage, three rRNA operons with identical 16S rRNA and slightly different 23S rRNA gene sequences are predicted. Phyla-AMPHORA (4) identified all 198 *Chloroflexi*-specific phylogenetic marker genes, which suggested that the draft genome is nearly complete and that the genome is comparable in size to those of other phototrophic *Chloroflexi* species (4.7 to 5.8 Mb [3]). Based on gene content and physiological studies, strain MS-G is an oxygen-tolerant anoxygenic phototroph.

Genes encoding type-2 (quinone-type) photosynthetic reaction centers (*pufLM*), and light-harvesting complex 1 (*pufAB*) as well as chlorosome proteins are present to enable phototrophic growth. The draft genome includes genes encoding all enzymes required for the synthesis of bacteriochlorophylls *a* and *c*, the presence of which was verified by high-performance liquid chromatography. A complete set of genes for the enzymes of the 3-hydroxypropionate cycle indicates the potential for autotrophic growth, which was observed in agar-shakes using sulfide-containing growth medium with bicarbonate and CO_2_ as the sole carbon sources in repeated transfers. The carotenoid biosynthesis pathway in strain MS-G is probably similar to that in other *Chloroflexus* spp., which produce γ-carotene, β-carotene, echinenone, 1′-OH-γ-carotene, and the glucoside and glucoside esters of 1′-OH-γ-carotene (5). Consistent with the production of these carotenoids, the MS-G genome contains phytoene synthase (*crtB*), homologs of phytoene saturases and desaturases (*crtI, crtD*), lycopene cyclase (*crtY*), a 1′, 2′hydratase (*cruF*), 1′-OH-glycosyltransferase (*cruC*), carotenoid glycoside acyltransferase (*cruD*), and beta-carotene 4-ketolase (*crtO*) (6, 7).

### Nucleotide sequence accession numbers.

This whole-genome shotgun project has been deposited at DDBJ/EMBL/GenBank under the accession JPIM00000000. The version described in this paper is version JPIM01000000.

## References

[1] Nübel U Bateson MM Madigan MT 2001 Diversity and distribution in hypersalinemicrobial mats of bacteria related to *Chloroflexus* spp. Appl. Environ. Microbiol. 67:4365–4371. 10.1128/AEM.67.9.4365-4371.2001 11526049 PMC93173

[2] Aziz RK Bartels D Best AA DeJongh M Disz T Edwards RA Formsma K Gerdes S Glass EM Kubal M Meyer F Olsen GJ Olson R Osterman AL Overbeek RA McNeil LK Paarmann D Paczian T Parrello B Pusch GD Reich C Stevens R Vassieva O Vonstein V Wilke A Zagnitko O 2008 The RAST server: Rapid Annotations using Subsystems Technology. BMC Genomics 9:75. 10.1186/1471-2164-9-75 18261238 PMC2265698

[3] Tang KH Barry K Chertkov O Dalin E Han CS Hauser LJ Honchak BM Karbach LE Land ML Lapidus A Larimer FW Mikhailova N Pitluck S Pierson BK Blankenship RE 2011 Complete genome sequence of the filamentous anoxygenic phototrophic bacterium *Chloroflexus aurantiacus*. BMC Genomics 12:334. 10.1186/1471-2164-12-334 21714912 PMC3150298

[4] Wang Z Wu M 2013 A phylum-level bacterial phylogenetic marker database. Mol. Biol. Evol. 30:1258–1262. 10.1093/molbev/mst059 23519313

[5] Takaichi S Tsuji K Matsuura K Shimada K 1995 A monocyclic carotenoid glucoside ester is a major carotenoid in the green filamentous bacterium *Chloroflexus aurantiacus*. Plant Cell Physiol. 36:773–778

[6] Maresca JA Graham JE Bryant DA 2008 The biochemical basis for structural diversity in the carotenoids of chlorophototrophic bacteria. Photosynth. Res. 97:121–140. 10.1007/s11120-008-9312-3 18535920

[7] Bryant DA Liu Z Li T Zhao F Costas AMG Klatt CG Ward DM 2012 Functional genomics and evolution of photosynthetic systems, p 47–102 *In* Burnap R Vermaas W (ed), Functional genomics and evolution of photosynthetic systems, advances in photosynthesis and respiration, vol 33 Springer Verlag, Dordrecht, Netherlands. 10.1007/978-94-007-1533-2

